# Direct effects of doxorubicin on skeletal muscle contribute to fatigue

**DOI:** 10.1038/sj.bjc.6604858

**Published:** 2009-01-13

**Authors:** K van Norren, A van Helvoort, J M Argilés, S van Tuijl, Karin Arts, M Gorselink, A Laviano, D Kegler, H P Haagsman, E M van der Beek

**Affiliations:** 1 Danone Research – Centre for Specialised Nutrition (formerly known as Numico Research), Wageningen, The Netherlands; 2 Cancer Research Group, Facultat de Biologia, Departament de Bioquímica i Biologia Molecular, Universitat de Barcelona, Barcelona, Spain; 3 Department of Clinical Medicine, University La Sapienza, Rome, Italy; 4 Faculty of Veterinary Medicine, Utrecht University, Utrecht, The Netherlands

**Keywords:** chemotherapy, animal models, quality of life

## Abstract

Chemotherapy-induced fatigue is a multidimensional symptom. Oxidative stress has been proposed as a working mechanism for anthracycline-induced cardiotoxicity. In this study, doxorubicin (DOX) was tested on skeletal muscle function. Doxorubicin induced impaired *ex vivo* skeletal muscle relaxation followed in time by contraction impediment, which could be explained by DOX-induced changes in Ca^2+^ responses of myotubes *in vitro*. The Ca^2+^ responses in skeletal muscle, however, could not be explained by oxidative stress.

Fatigue, defined as sustained exhaustion and decreased functional capacity not relieved by rest, is one of the most common side effects of chemotherapy in cancer patients. Chemotherapy-induced fatigue has been associated with asthenia (Morrow *et al*, 2002). Cardiomyopathy is a major adverse effect in patients treated with anthracyclines. One mechanism described, explaining the cardiotoxicity of doxorubicin (DOX) in cardiomyopathy, is an increase in oxidative stress due to a decrease in glutathione concentrations (Wallace, 2007). This increase in oxidative stress subsequently leads to a loss of mitochondrial integrity and function, eventually leading to an increase in calcium concentrations leaking from the mitochondria. Positive results in reducing cardiotoxicity have been reported when antioxidants were used in combination with DOX. However, results obtained are not completely conclusive (Singal *et al*, 1997). It has been described that DOX is extruded from the cardiac myocyte conjugated to GSH by multidrug resistance proteins (MRPs) (Krause *et al*, 2007). Extensive extrusion of DOX subsequently induces GSH depletion. This DOX-induced imbalance of the redox status supposedly induces mitochondrial permeability leading to changes in calcium fluxes as observed in DOX-induced cardiotoxicity. In this study, the effect of DOX on skeletal muscle was studied as well as the effect of oxidative stress on this process.

## Materials and methods

Contractile characteristics of the extensor digitorum longus (EDL) from male CD2F1 mice, 7–9 weeks old, were assessed *ex vivo* as described earlier (Gorselink *et al*, 2006). Muscles were incubated (0–120 min) with a DOX concentration range of 50–175 *μ*M, being below maximal plasma concentrations reached in clinical practice (Delgado *et al*, 1989). In differentiated C2C12 myotubes (obtained after 6 days of culturing in DMEM+0.4% UltroserG), calcium responses were measured fluorescently (FURA-2 AM ester). Effect of overnight incubation with DOX (0–10 *μ*M), electron chain inhibitors (rotenone 0.1–10 *μ*M or piericidin A 0.25–2.5 *μ*M) and antioxidants (*N*-acetyl cysteine (NAC) 10 *μ*M–1 mM or trolox 10 *μ*M–1 mM) was studied.

## Results

**Figure 1:**
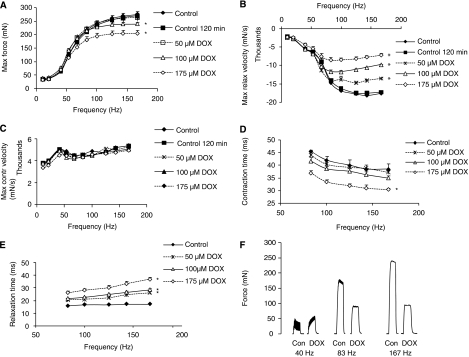
Effects of doxorubicin (DOX) on muscle function of mouse EDL. (**A**–**E**) Incubations were carried out for 1 h unless stated otherwise. (**A**) Maximal force. (**B**) Maximal relaxation velocity. (**C**) Maximal contraction velocity. (**D**) CT at tetanus. (**E**) RT at tetanus. (**F**) Typical examples of contraction curves after 120 min of incubation with 175 *μ*M DOX. Data: mean±s.e.m. ^*^Adjacent to curve (for complete curve): *P*<0.05 *vs* control.

**Figure 2:**
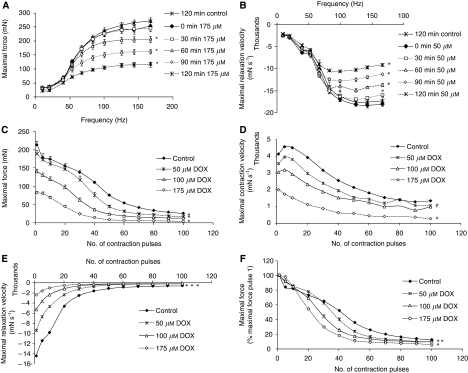
Effects of doxorubicin (DOX) on contractile function of mouse EDL. (**A**) Maximal force (175 *μ*M DOX for 0–2 h). (**B**) Maximal relaxation velocity (50 *μ*M DOX for 0–2 h). (**C**–**E**) Effects of DOX on repetitive stimulation on maximal force, maximal contraction and relaxation velocity, respectively. (**F**) Effect of DOX on maximal force corrected for maximal force at the start of the repetitive stimulation. Data: mean±s.e.m. ^*^Above point or adjacent to curve (for complete curve): *P*<0.05 *vs* control.

Contractile characteristics of mouse EDL remained stable during the complete incubation period: control curves at *t*=0 and *t*=120 min were identical for all parameters measured (Figures 1A–C, 2A and B). None of the DOX concentrations used led to LDH release. Reductions in maximal force, contraction or relaxation velocity remained stable in time, if muscles were rinsed for 2 h subsequently.

Incubation with 100 or 175 *μ*M DOX for 1 h or more resulted in a time- and concentration-dependent decrease of maximal forces (Figures 1A, 2A and C). Maximal relaxation velocity was already affected at lower concentrations (⩾50 *μ*M DOX) and after a shorter incubation period (⩾0.5 h) (Figures 1B and 2B). The maximal contraction velocity (Figure 1C) decreased after a 1.5 h incubation with 100 *μ*M DOX (Figure 2E, first contraction). After 1 h incubation, CT (contraction time required to increase the force from 10 to 90% of the maximal force) was proportional to the maximal force (Figure 1D). RT (relaxation time from 90 to 10% of the maximal force), however, was longer, implying impaired relaxation (Figure 1E). In the presence of DOX, at low frequencies (40 Hz), maximal force increased during the contraction phase, whereas in the controls, force remained constant or slightly decreased (1F). This difference in shape of the curve seems to disappear at higher frequencies. So, the sequence of events induced by the exposure of skeletal muscle to DOX starts with an attenuated maximal relaxation velocity, followed by a decreased maximal force and a longer relaxation time.

To estimate the effect of DOX on fatigue, a moderate exercise protocol (83 Hz, 250 ms every 1000 ms, 100 contractions) was performed. Stimulation after 2 h of incubation with DOX resulted in impaired maximal force, contraction and relaxation velocity (Figure 2C–E) at the start of the exercise protocol. These differences remained and further increased during the repetitive stimulations (Figure 2E and F).

**Figure 3:**
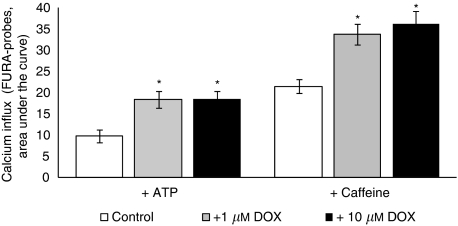
Calcium response of C2C12 myotubes o/n incubated with DOX, measured immediately after stimulation with ATP or Caffeine. Data: mean +/− s.e.m.

These results indicate that incubations with DOX result in concentration- and time-dependent decreases in muscle performance both during single and repetitive contraction pulses. Again, the sequence of events starts off with an impaired muscle relaxation velocity. Relaxation velocity is directly related to the rate of clearance of calcium from the cytoplasm (MacLennan, 2000; Pan *et al*, 2003). C2C12 myotubes were used as an *in vitro* model to examine if DOX affected Ca^2+^ fluxes. An overnight incubation with DOX resulted in increased calcium responses to ATP and caffeine (Figure 3).

To test the magnitude of the effect of oxidative stress on changes in calcium influx, oxidative stress was induced in C2C12 cells (Table 1). Free radicals were generated by inducing malfunction of the mitochondria through the addition of the respiratory chain inhibitor rotenone or piericidin A. Despite the fact that these components induced toxicity resulting in cell death at higher concentrations, addition of none of these components resulted in an increased calcium influx in the C2C12 cells at concentrations not affecting cell vitality. Moreover, the addition of trolox, a water-soluble vitamin E antioxidant, effective in both the lipid and the water phase, did not result in a reduction of the DOX-induced increase in calcium influx in the C2C12 cell system. In addition, incubations with NAC did not result in the inhibition of the increased calcium influx.

## Discussion

The physiological aspects of cancer-related fatigue are multifactorial and are both tumour and therapy related (Morrow *et al*, 2002). A poor nutritional status, cancer cachexia and chemotherapy-induced fatigue have been associated with muscle weakness and reduced quality of life (Morrow *et al*, 2002; Gorselink *et al*, 2006). This paper shows that DOX can directly impair skeletal muscle relaxation, followed in time by defective contraction. Doxorubicin has been described for heart muscle to interfere with the respiratory chain (Ishikawa *et al*, 2006) and to inhibit oxidative phosphorylation. This altered mitochondrial function is believed to induce increased oxidative stress in the mitochondria (Wallace, 2007), leading to malfunction of the mitochondria. Shortage in energy would then cause leakage of calcium from the mitochondria into the cytoplasm. Mimicking this effect, however, with two different respiratory chain inhibitors did not result in increased calcium influxes in the C2C12 cell system. The addition of different kinds of antioxidants also did not reduce the DOX-induced hypercalcaemia. Controversies in the results obtained for studies testing different antioxidants in preventing cardiomyopathy have been suggested to be caused by differences in the ability of the antioxidants to be effective in both the lipid and the water phase (Singal *et al*, 1997). Therefore, trolox, an antioxidant active in both the water and the lipid phase, was tested. However, it did not reduce the DOX-induced increase in calcium response. Alternatively, DOX has been described to reduce GSH levels due to an extensive extrusion of GSH-conjugated DOX by MRPs (Krause *et al*, 2007). *N*-acetyl cysteine supplementation has been described to be capable of restoring GSH stores (Kelly, 1998). However, the incubation of C2C12 cells with NAC did not decrease the DOX-induced calcium influx. Krause *et al* (2007) described a marked difference in the expression of the MRP1/GS-X pump between skeletal muscle and cardiac muscle. Although the cardiomyocytes show an abundant expression of this pump, expression is completely absent in skeletal muscle cells. MRP1/GS-X is one of the two MRP proteins involved in extruding DOX conjugated with glutathione from the cytoplasm in cardiomyocytes. RLIP76 (RALBP1), the other GS-X pump involved in the transport of GSH-conjugated DOX out of the heart muscle, seems to be expressed 1.5 times more in heart muscle compared with skeletal muscle (Awasthi *et al*, 2008). A marked difference in the expression of MRPs between skeletal and cardiac muscle might be an explanation for the differences in the reaction to antioxidants observed. Next to that, Lebrecht *et al* (2003) reported that in DOX-treated rats, activities of enzymes of the respiratory chain were decreased in cardiac muscle but not in skeletal muscle. We therefore hypothesise that in skeletal muscle, the increased calcium influx induced by DOX is not due to radical induced malfunction of the mitochondria.

In (skeletal) muscle, Ca^2+^ can be released by the depolarisation or by the activation of the purigenic receptor by ATP. In case of depolarisation, the dihydropyridine receptor, a voltage-sensitive Ca^2+^ channel, interacts with the ryanodine receptor (RyR) to release Ca^2+^ from the sarcoplasmic reticulum (SR). Caffeine can activate the RyR directly. If the purigenic receptor is stimulated by ATP, IP3 is formed, which in turn releases Ca^2+^ from the SR. Ca^2+^-induced Ca^2+^ release can then reinforce RyR stimulation. The elevated Ca^2+^ can be redistributed to mitochondria through the Ca^2+^ uniporter or to the SR through SERCA pump. Data from skeletal muscle SR fragments indicated that DOX could bind to Ryr-1 resulting in Ca^2+^ release from the SR (Abramson *et al*, 1988). However, we could not detect a spontaneous DOX-induced Ca^2+^ flux in the absence of a contraction-stimulating component like ATP or caffeine. The difference between these findings might be that the C2C12 myotubes are an intact cellular system, whereas Abramson *et al* (1988) used SR fragments. Our finding that relaxation time is especially changed in skeletal muscle suggests a reduction in Ca^2+^ re-uptake in the mitochondria or SR, leading initially to increased calcium responses and prolonged muscle relaxation times. But a decrease in the velocity of Ca^2+^ re-uptake leading to increased intracellular calcium levels might, however, lead to an increase of the maximal force during the contraction phase (MacLennan, 2000). This effect is indeed present at a lower frequency, resulting in a different shape of the contraction curve (Figure 1F, 40 Hz). These data are in line with the findings of De Beer *et al* (1992) showing that the sensitivity of skeletal muscle to extracellular calcium increases in DOX-treated skinned single skeletal muscle fibres. At higher frequencies, however, maximal force did not further rise during the contraction (Figure 1F). This might be due to depleted Ca^2+^ stores due to a restrained Ca^2+^ re-uptake in the SR. This aligns with data showing that DOX-induced cardiac dysfunction is associated with a lack rather than an excess of calcium (Jensen, 1986) and with SERCA-1 knockouts showing a depressed maximal force output (Pan *et al*, 2003). A prolonged exposure to DOX might lead to an increased apoptosis due to the initial Ca^2+^ overload, whereas the surviving skeletal muscle cells might suffer from emptied Ca^2+^ stores due to a continuous reduction of Ca^2+^ re-uptake. The DOX-induced, oxidative stress-independent changes in skeletal muscle function might also be present in cardiac muscle, but overlooked because of the presence of oxidative stress-induced side effects. Anthracyclines are widely used to treat solid tumours and haematological malignancies (Hortobagyi, 1997). Patients suffering from these types of cancers are also at risk of developing hypercalcaemia (Higdon and Higdon, 2006). Therefore, this patient group might even be more susceptible to sustained exhaustion as a result of impaired muscle function due to chemotherapy-induced subcellular calcaemic disturbances.

## Figures and Tables

**Table 1:** The effect of antioxidants and respiratory chain inhibitors on calcium influx in C2C12 cells

**Calcium influx in C2C12 cells**	**Mean±s.e.m.**	***P*-value**
**Addition of antioxidants**		
10 *μ*M doxorubicin	1.97±0.14	
10 *μ*M doxorubicin+1 mM trolox	2.40±0.45	0.2
10 *μ*M doxorubicin+0.5 mM trolox	2.11±0.14	0.5
10 *μ*M doxorubicin+0.1 mM trolox	2.43±0.11	0.2
10 *μ*M doxorubicin+10 *μ*M trolox	2.13±0.44	0.6
10 *μ*M doxorubicin+1 mM *N*-acetyl cysteine	1.95±0.18	1.0
10 *μ*M doxorubicin+0.1 mM *N*-acetyl cysteine	2.10±0.10	0.7
10 *μ*M doxorubicin+10 *μ*M *N*-acetyl cysteine	1.84±0.36	0.7
		

**Addition of respiratory chain inhibitors**	**Mean±s.e.m.**	***P*-value**
10 *μ*M rotenone	0.81±0.15	0.001
0.1 *μ*M rotenone	0.69±0.08	0.000
25 *μ*M piericidin A	0.92±0.23	0.001
2.5 *μ*M piericidin A	0.74±0.03	0.000
0.25 *μ*M piericidin A	0.91±0.10	0.001

Data are expressed relative to control values without doxorubicin (DOX). *P*-values are expressed as compared with values with 10 *μ*M DOX present. None of the antioxidants added resulted in a significant decrease in DOX-induced calcium influx (no significant difference with DOX alone). None of the respiratory chain inhibitors resulted in an increase in calcium influx (all values below 1). Therefore, all the values were significantly different from incubations with 10 *μ*M DOX.
