# Direct effects of doxorubicin on skeletal muscle contribute to fatigue

**DOI:** 10.1038/sj.bjc.6604992

**Published:** 2009-03-17

**Authors:** K van Norren, A van Helvoort, J M Argiles, S van Tuijl, K Arts, M Gorselink, A Laviano, D Kegler, H P Haagsman, E M van der Beek

**Correction to**: British Journal of Cancer (2009) **100**, 311–314; doi:10.1038/sj.bjc.6604858

On correction of the above article, the affiliations of some of the authors were incorrectly presented. The correct affiliations are as follows:

K van Norren^1,2,7^, A van Helvoort^1,7^, JM Argilés^3^, S van Tuijl^1^, K Arts^1^, M Gorselink^4^, A Laviano^5^, D Kegler^1^, HP Haagsman^6^ and EM van der Beek^1^

^1^Danone Research – Centre for Specialised Nutrition (formerly known as Numico Research), Wageningen, The Netherlands; ^2^Nutrition and Pharmacology Group, Division of Human Nutrition, Wageningen University, Wageningen, The Netherlands; ^3^Cancer Research Group, Facultat de Biologia, Departament de Bioquímica i Biologia Molecular, Universitat de Barcelona, Barcelona, Spain; ^4^Center for Innovative Consumer Studies, Wageningen UR, Wageningen, The Netherlands; ^5^Department of Clinical Medicine, University La Sapienza, Rome, Italy; ^6^Faculty of Veterinary Medicine, Utrecht University, Utrecht, The Netherlands

^7^These authors contributed equally to this work.

